# Bridging East and West: Real-World Clinicogenomic Landscape of Metastatic NSCLC in Türkiye

**DOI:** 10.3390/genes16121446

**Published:** 2025-12-03

**Authors:** Kübra Canaslan, Emre Eken, Mehmet Bilici, Fahriye Merve Balcıoğlu, Banu Öztürk, Mehmet Çakmak, Öznur Bal, Görkem Turhan, Feyyaz Özdemir, Hayati Arvas, Zuhat Urakçı, Ebru Çiçek, Zeynep Hande Turna, Aziz Karaoğlu

**Affiliations:** 1 Department of Medical Oncology, Dokuz Eylul University, Izmir 35330, Türkiye; 2 Department of Medical Oncology, Atatürk University, Erzurum 25240, Türkiye; 3 Department of Medical Oncology, Antalya Research and Training Hospital, Antalya 07100, Türkiye; 4 Department of Medical Oncology, Bilkent City Hospital, Ankara 06800, Türkiye; 5 Department of Medical Oncology, Karadeniz Technical University, Trabzon 61080, Türkiye; 6 Department of Medical Oncology, Dicle University, Diyarbakır 21280, Türkiye; 7 Department of Medical Oncology, İstanbul University Cerrahpaşa, Istanbul 34320, Türkiye

**Keywords:** non-small cell lung cancer, Genomics, Next-generation sequencing

## Abstract

Background/Objectives: Genomic profiling guides treatment in metastatic non-small-cell lung cancer (mNSCLC), yet country-level data from Türkiye remain limited. Methods: We retrospectively analyzed consecutive patients with mNSCLC diagnosed between January 2018 and March 2025 across tertiary centers in all seven regions. Variables included demographics, smoking, histology, testing modality (single-gene vs. next-generation sequencing [NGS]), targetable genomic alterations (TGAs) and co-mutations, and programmed death-ligand 1 (PD-L1) tumor proportion score. Results: Among 1023 patients (mean age 64 years; 76.4% male), tobacco exposure was frequent (mean 42.1 pack-years); 16.9% were never-smokers. NGS use increased over time, exceeding 90% by 2025. TGAs were detected in 28.3% (EGFR 16.0%, ALK 5.0%, KRAS G12C 2.6%, BRAF V600E 3.2%; ROS1, MET exon 14, HER2, NTRK ≤ 2.5%; no RET). EGFR alterations occurred in 19% of non-squamous carcinomas and 6% of squamous cell carcinomas (SCCs), suggesting an intermediate East–West pattern. Among NGS-tested samples, TP53 was the most frequent co-mutation (33.1%), followed by alterations in CDKN2A, PIK3CA, FGFR, STK11, and KEAP1. Conclusions: In this large, multicenter Turkish real-world cohort, the TGA spectrum broadly mirrors global patterns while revealing local nuances; EGFR mutations were more frequent than expected in SCC, and nationwide NGS adoption is accelerating. Limitations include retrospective design, non-centralized PD-L1 testing, and missing data. Prospective, standardized studies integrating outcomes and resistance mechanisms are warranted to refine regional precision oncology.

## 1. Introduction

Cancer is a genetic disease driven by oncogenic and tumor-suppressor alterations that rewire proliferation, survival, genomic stability, and immune evasion. Over the last century, treatment evolved from surgery, radiation, and cytotoxic chemotherapy to molecularly targeted agents and immunotherapies, reshaping outcomes in biomarker-defined subsets. Molecular profiling is therefore no longer optional background—it is the mechanism by which actionable drivers are found, resistance is understood, and immunotherapy biomarkers are interpreted in context [1].

Within thoracic oncology, non-small-cell lung cancer (NSCLC) constitutes the majority of lung cancer cases and is characterized by significant histological and molecular heterogeneity. Over the past two decades, the treatment paradigm has shifted from empiric platinum-based chemotherapy to increasingly precise strategies driven by tumor biology [2]. This evolution, which centers on identifying targetable genomic alterations (TGAs) (e.g., *EGFR*, *ALK/ROS1*, *BRAF V600E*, *MET exon 14*, *RET*, *NTRK*, *HER2*) and immune markers like programmed death-ligand 1 (PD-L1), has reshaped therapeutic decision-making globally [3]. While the rise of molecular diagnostics and targeted/immunotherapies have reshaped treatment paradigms, the available genomic data predominantly derive from Western or East Asian populations. This disparity underscores the need for real-world data from diverse populations to ensure regional applicability of precision medicine standards. In parallel, molecular testing has moved from single-gene assays to broad next-generation sequencing (NGS), reshaping diagnostic workflows and therapeutic decision-making [4].

Türkiye, with its population of approximately 85 million, located at the crossroads of Europe and Asia, represents a unique population in terms of both genetic diversity and environmental exposures [5]. In Türkiye, the life expectancy at birth for the 2022–2024 period was approximately 78.1 years, with an average of about 80.7 years for women and 75.5 years for men [6]. Türkiye currently holds the highest lung cancer incidence rate among men globally, where it remains the most frequently diagnosed malignancy and the leading cause of cancer-related death [7]. This high burden is largely attributed to the country’s elevated tobacco consumption and associated risk factors [8].

Given this context, country-level data from Türkiye remains limited. This multicenter retrospective study aimed to characterize the demographic, histological, and molecular features of patients with metastatic NSCLC (mNSCLC) in Türkiye. In particular, it aimed to assess the distribution of actionable genomic alterations and programmed death-ligand 1 (PD-L1) expression and explore their associations with smoking history.

## 2. Methods

This retrospective, multicenter cohort study was conducted across seven geographical regions of Türkiye, including Marmara, Aegean, Mediterranean, Central Anatolia, Black Sea, Eastern Anatolia, and Southeastern Anatolia. The highest-volume tertiary center specializing in cancer care and molecular testing contributed data from each region. A total of 1023 patients diagnosed with mNSCLC from January 2018 to March 2025 were included. Patients were eligible if they had histologically confirmed mNSCLC, stage IV at diagnosis or metastatic relapse, and available molecular profiling data, regardless of histologic subtype or line of therapy. Patients with tumors with neuroendocrine or small cell features, limited molecular data (less than three genes), and non-metastatic disease were excluded.

Demographic data (age, sex), smoking history (pack-years and smoking status), histological subtype, and clinical presentation (de novo vs. recurrent metastatic disease) were recorded. Molecular testing information included the method of testing [single-gene polymerase chain reaction (PCR)-based assays or next-generation sequencing (NGS)], identified genomic alterations (e.g., epidermal growth factor receptor (*EGFR*), anaplastic lymphoma kinase (*ALK*), Kirsten rat sarcoma viral oncogene homolog (*KRAS*), ROS proto-oncogene 1 (*ROS1*), B-Raf proto-oncogene V600E mutation (*BRAF* V600E), mesenchymal–epithelial transition exon 14 skipping mutation (*MET* exon 14 skipping), rearranged during transfection (*RET*), human epidermal growth factor receptor 2 (*HER2*), neurotrophic tyrosine receptor kinase (*NTRK*)), and co-mutations (e.g., tumor protein p53 (*TP53*), serine/threonine kinase 11 (*STK11*), Kelch-like ECH-associated protein 1 (*KEAP1*), and phosphatidylinositol-4,5-bisphosphate 3-kinase catalytic subunit alpha (*PIK3CA*)). Single-gene testing and NGS were performed locally at participating tertiary centers using their routine, validated assays. *ALK* and *ROS* rearrangements were identified by fluorescent in situ hybridization (FISH). Because NGS panels and vendors differed across sites, we harmonized outputs with a predefined list of targetable genomic alterations and excluded variants of uncertain significance (VUSs) from driver/co-mutation counts. For analysis, alterations were coded as present/absent per patient level using Human Genome Variation Society (HGVS) nomenclature on a common build; no attempt was made to harmonize panel size, depth, or bioinformatic pipelines. Molecular frequencies are reported as “positive/N tested”.

PD-L1 expression was evaluated by immunohistochemistry (IHC) in local pathology laboratories using guideline-concordant, vendor-approved assays and reported as tumor proportion score (TPS) in three categories, <1%, 1–49%, and ≥50%, per local protocol. PD-L1 positivity was defined as PD-L1-TPS ≥ 1%. Since assays and platforms were not centralized, PD-L1 analyses are considered exploratory. Complete site-level specifications for NGS panels (genes, variant classes, LOD, depth, and pipelines) and PD-L1 assays (clone/platform, scoring, adoption year) are provided in Supplementary Table S1.

Adenocarcinoma, adenosquamous carcinoma, and non-small-cell carcinoma not otherwise specified are reported as non-squamous carcinoma. Targetable genomic alterations (TGA) were defined as *EGFR*, *ALK*, *KRAS G12C*, *ROS1*, *BRAF* V600E, *MET* exon 14 skipping, *RET*, *HER2*, and *NTRK*. Treatment data included only the number of systemic therapy lines received. Overall survival (OS) was defined as the time from diagnosis of metastatic disease to death or last follow-up.

All statistical analyses were performed using IBM SPSS Statistics version 29.0 (IBM Corp., Armonk, NY, USA) and R version 4.5.2 (R Foundation for Statistical Computing, Vienna, Austria). Descriptive statistics were used to summarize demographic and clinical characteristics. When appropriate, categorical variables were presented as frequencies and percentages and compared using the Pearson chi-square test or Fisher’s exact test. Continuous variables were expressed as mean ± standard deviation or median (range), depending on the distribution assessed by the Kolmogorov–Smirnov test. Associations between genomic alterations and clinicopathologic parameters, including age group, sex, smoking status, histology, and PD-L1 expression, were evaluated using cross-tabulations. Associations between driver alterations and PD-L1 positivity were assessed using multivariable logistic regression adjusted for age, sex, smoking status, histologic subtype, and PD-L1 assay. Two-sided *p*-values were corrected for multiple testing using the Benjamini–Hochberg false discovery rate (FDR) procedure. OS was estimated using the Kaplan–Meier method. Median survival times were reported with corresponding 95% confidence intervals (CIs). Statistical significance was defined as a two-sided *p*-value of less than 0.05.

## 3. Results

### 3.1. Patient Characteristics

A total of 1023 patients with mNSCLC were included in the study. The mean age at diagnosis was 64.0 ± 9.8 years, and 49.4% of the patients were aged ≥65 years. The majority were male (76.4%) and had a history of tobacco exposure, with a mean cumulative smoking index of 42.1 ± 28.8 pack-years. Never-smokers comprised only 16.9% of the cohort. Most patients presented with de novo metastatic disease (75.2%), while the remainder had recurrent metastasis after prior curative treatment (24.8%).

### 3.2. Molecular Testing

**Figure 1:**
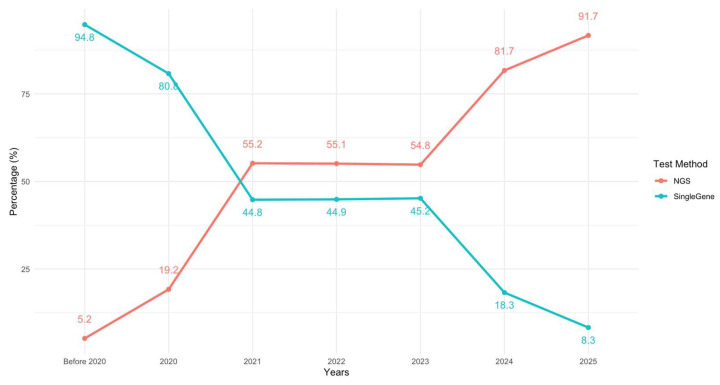
Temporal trends in molecular testing methods for NSCLC: single-gene testing vs. NGS (before 2020–2025). NGS: next-generation sequencing.

Molecular profiling was performed using NGS in 528 patients (51.6%) and single-gene assays in 495 patients (48.4%). The use of NGS increased significantly over time, rising from only 5.2% of molecular tests in 2020 and earlier to more than 90% in 2025, indicating a substantial shift toward comprehensive genomic profiling in recent years (Figure 1). The center-level year-by-year distribution is shown in Supplementary Table S2.

### 3.3. Driver Mutations

**Figure 2:**
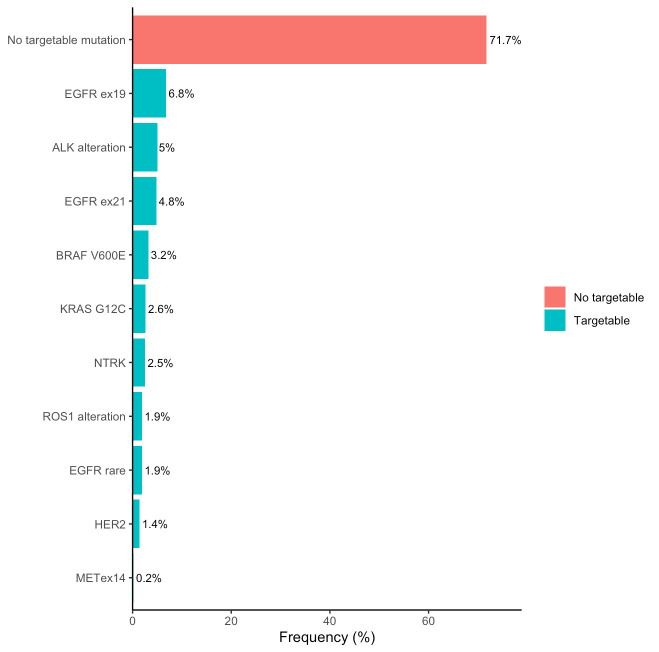
Distribution of targetable genomic alterations in metastatic NSCLC. Because some patients harbored > 1 actionable alteration (n = 10, 1.0%), they were counted in the overall total and in each relevant gene slice (non-mutually exclusive). Consequently, slice percentages may exceed 100%. Percentages reflect gene-level frequencies. *ALK*: anaplastic lymphoma kinase, *BRAF*: B-Raf proto-oncogene, *EGFR* ex19: epidermal growth factor receptor exon 19 deletions, *EGFR* ex21: epidermal growth factor receptor exon 21 mutation, *HER2:* human epidermal growth factor receptor 2, *KRAS*: Kirsten rat sarcoma viral oncogene homolog, *MET*ex14: mesenchymal–epithelial transition exon 14 skipping mutation, *NTRK*: neurotrophic tyrosine receptor kinase, *ROS1*: ROS proto-oncogene 1.

Targetable genomic alterations were identified in 289 patients (28.3%). The most frequently detected alterations included *EGFR* mutations in 164 patients (164/1023; 16.0%), with exon 19 deletions (6.8%) and L858R point mutations in exon 21 (4.8%) representing the most common subtypes. Rare *EGFR* mutations were present in 1.9%, and *EGFR* exon 20 insertions were observed in 0.9%. Other targetable alterations included *ALK* rearrangements in 5.0% (51/992), *KRAS* G12C mutations in 2.6% (26/990), *ROS1* fusions in 1.9% (18/992), *BRAF* V600E in 3.2% (22/693), *HER2* mutations in 1.4% (8/552), *MET* exon 14 skipping in 2.5% (13/527), and *NTRK* fusion in 0.2% (1/504). No *RET* rearrangements were identified in the cohort (0/525) (Figure 2). In total, 10 patients (1.0%) harbored dual actionable mutations. These included *EGFR* with *BRAF* V600E (n = 2), *BRAF* V600E with *KRAS* G12C (n = 1), *ROS1* with *BRAF* V600E (n = 1), *ROS1* with *EGFR* (n = 1), *EGFR* with *MET* exon 14 skipping (n = 2), *KRAS* G12C with *MET* exon 14 skipping (n = 1), *KRAS* G12C with *NTRK* fusion (n = 1), and *ALK* with *BRAF* V600E (n = 1).

### 3.4. Frequency of Targetable Mutations by Histologic Subtype

**Figure 3:**
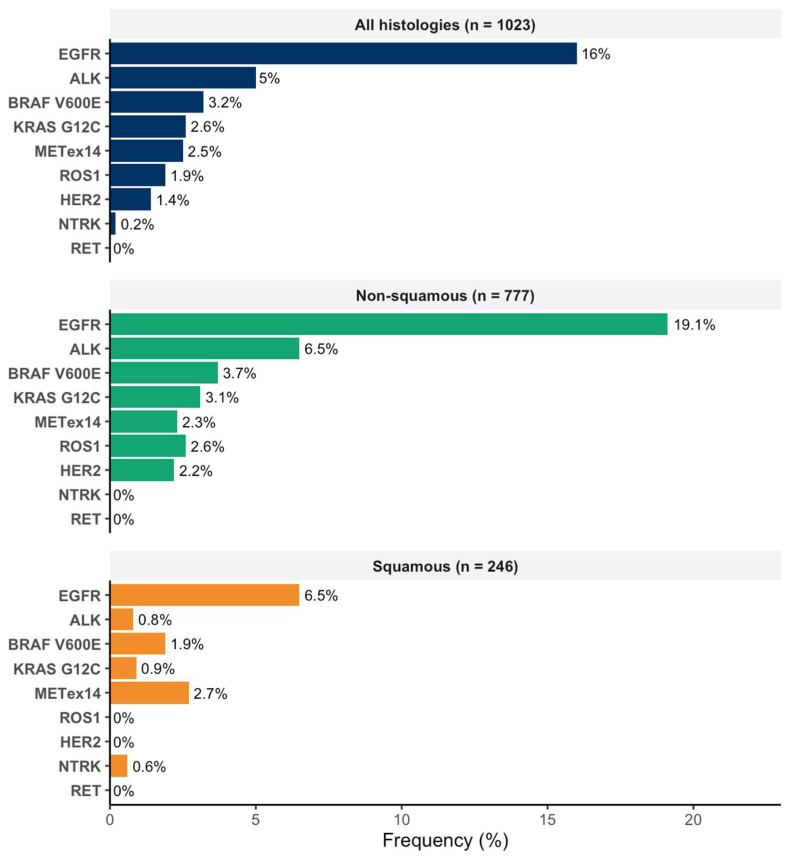
Frequency of targetable genomic alterations by histologic subtype in metastatic NSCLC. *ALK*: anaplastic lymphoma kinase, *BRAF*: B-Raf proto-oncogene, *EGFR* ex19: epidermal growth factor receptor exon 19 deletions, *EGFR* ex21: epidermal growth factor receptor exon 21 mutation, *HER2*: human epidermal growth factor receptor 2, *KRAS*: Kirsten rat sarcoma viral oncogene homolog, *MET*ex14: mesenchymal–epithelial transition exon 14 skipping mutation, *NTRK*: neurotrophic tyrosine receptor kinase, *ROS1*: ROS proto-oncogene 1.

When stratified by histologic subtype, *EGFR* mutations were significantly more frequent in non-squamous tumors (19.1%) than in squamous cell carcinomas (6.5%). Similarly, *ALK* rearrangements were more common in non-squamous histology (6.5%) than in squamous tumors (0.8%). *ROS1* fusions were detected in 2.6% of non-squamous and 1.9% of squamous carcinomas, while *BRAF* V600E mutations were found in 3.7% and 1.9%, respectively. *KRAS* G12C mutations occurred in 3.1% squamous and 2.6% non-squamous tumors. *HER2* mutations were also slightly more common in non-squamous histology (2.2%) than in squamous histology (1.4%). *MET* exon 14 skipping mutations were relatively evenly distributed across both histologic subtypes (2.5% in non-squamous vs. 2.3% in squamous). *NTRK* fusions were rare overall (0.2%) and not observed in squamous tumors. No *RET* rearrangements were identified in either group (Figure 3).

### 3.5. PD-L1 Expression and Association with Oncogenic Driver Mutations

In exploratory analyses, PD-L1-TPS was <1% in 47.8%, between 1 and 49% in 34.4%, and ≥50% in 17.8% of 601 patients with available data. EGFR-mutated tumors were more frequently associated with lower PD-L1 expression compared to EGFR wild-type tumors. A total of 42.7% (n = 38) of *EGFR*-mutated tumors were PD-L1-positive, whereas 54.2% (n = 276) of *EGFR* wild-type tumors expressed PD-L1. *ROS1*-rearranged tumors (n = 11) demonstrated a lower PD-L1 positivity rate (18.2%, n = 2) compared to *ROS1*-negative tumors (53.8%, n = 304), a statistically significant difference (*p* = 0.019), although the number of *ROS1*-positive cases was relatively small. Multivariable logistic regression (adjusted for age, sex, smoking, histology, and site) followed by Benjamini–Hochberg FDR correction across drivers showed no statistically significant associations between PD-L1 positivity (TPS ≥ 1%) and *EGFR*, *ALK*, *ROS1*, *KRAS* G12C, or *BRAF* V600E (all q ≥ 0.29) (Supplementary Table S3).

No meaningful associations were found between PD-L1 status and the presence of other alterations, including *ALK* (52.0% vs. 52.6%, *p* = 0.956), *BRAF* V600E (46.2% vs. 52.7%, *p* = 0.642), *KRAS* G12C (40.0% vs. 52.5%, *p* = 0.271), *HER2* (66.7% vs. 52.3%, *p* = 0.484), *MET* exon 14 (0.0% vs. 51.7%, *p* = 0.302), and *NTRK* (0.0% vs. 53.1%, *p* = 0.288), suggesting that PD-L1 expression is largely independent of these oncogenic alterations in this cohort.

### 3.6. Co-Mutations in NGS-Tested Patients

Among 520 patients who underwent NGS testing with the optimal level of gene coverage, the most frequent co-mutations were *TP53* (33.1%), *CDKN2A* (4.1%), *PIK3CA* (3.2%), *FGFR* (3.0%), *STK11* (2.6%), *KEAP1* (2.0%), *RB1* (2.0%), *NF1* (2.0%), *PTEN* (1.5%), *ARID1* (1.9%), and *ATRX* (0.9%).

**Figure 4:**
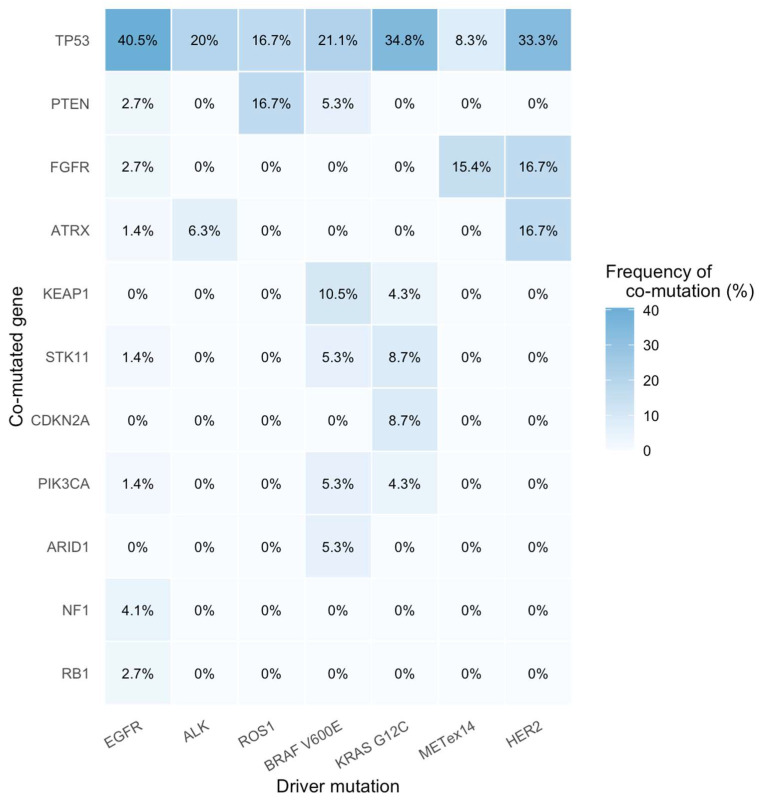
Distribution of co-mutations in patients with targetable genomic alterations. *ALK:* anaplastic lymphoma kinase, *ARID1*: AT-rich interaction domain 1A, *ATRX*: Alpha Thalassemia/Mental Retardation Syndrome X-linked, *BRAF*: B-Raf proto-oncogene, *CDKN2A*: cyclin-dependent kinase inhibitor 2A, *EGFR*: epidermal growth factor receptor, *FGFR*: fibroblast growth factor receptor, *HER2*: human epidermal growth factor receptor 2, *KEAP1*: Kelch-like ECH-associated protein 1, *KRAS*: Kirsten rat sarcoma viral oncogene homolog, *MET*ex14: mesenchymal–epithelial transition exon 14 skipping mutation, *NF1*: Neurofibromatosis type 1, *PIK3CA*: phosphatidylinositol-4,5-bisphosphate 3-kinase catalytic subunit alpha, *PTEN*: Phosphatase and TENsin homolog, *RB1*: Retinoblastoma 1, *ROS1*: ROS proto-oncogene 1, *STK11*: serine/threonine kinase 11, *TP53*: Tumor protein p53.

Among patients harboring targetable driver alterations, *TP53* was the most frequent co-mutation across multiple subgroups, observed in 40.5% of *EGFR*-mutant, 20.0% of *ALK*-rearranged, 34.8% of *KRAS* G12C-mutant, 33.3% of *HER2-*mutant, 21.1% of *BRAF* V600E-mutant, 16.7% of *ROS1*-rearranged, and 8.3% of *MET* exon 14 skipping cases. Other notable co-mutations included *STK11* in *KRAS* G12C (8.7%) and *BRAF* V600E (5.3%); *KEAP1* in *BRAF* V600E (10.5%), *KRAS* G12C (4.3%); *PTEN* in *ROS1* (16.7%) and *EGFR* (2.7%); *FGFR* alterations in *MET* exon 14 skipping (15.4%) and *HER2* (16.7%). *ARID1* mutations were uncommon, present only in *BRAF* V600E (5.3%) (Figure 4).

### 3.7. Associations Between Clinical Characteristics and Driver Mutations

*EGFR*-mutated patients were more often female, never-smokers, and had non-squamous histology compared to their wild-type counterparts. *ALK* fusion-positive tumors were also enriched in younger, female, never-smokers with non-squamous histology. *ROS1* rearrangements occurred predominantly in younger, female, never-smokers with non-squamous histology, similar to the *ALK* pattern. *BRAF* V600E-mutated tumors shared the tendency toward non-squamous histology but did not differ significantly from wild-type cases in other demographic or clinical features. Comparative distributions of age, sex, smoking status, histologic subtype, and PD-L1 expression across these molecular subgroups are presented in Table 1.

### 3.8. Treatment and Follow-Up

The median follow-up duration for the study cohort was 29.1 months, and the median OS was 14.4 months. The median number of systemic therapy lines received was 2 (range, 0–8). Forty-two patients did not receive any systemic treatment due to poor performance status or treatment refusal, and two patients received only local treatment to metastatic sites. A total of 978 patients received at least one line of systemic therapy.

## 4. Discussion

This multicenter retrospective study provides a real-world landscape of genomic alterations and PD-L1 expression patterns among patients with mNSCLC across Türkiye. Beyond describing mutation frequencies, our data capture how molecular testing is currently implemented in routine practice, including heterogeneity in testing strategies. The diversity of genomic profiles observed in this Turkish population at the crossroads of Europe and Asia underscores the need to adapt global precision oncology standards to regional realities, with implications for test selection and for planning future access and policy discussions. Consistent with regional studies investigating genetic variants in other malignancies, such as genomic variants in bladder cancer and genomic disparity in breast cancer patients from Türkiye [9,10], our findings reinforce that regional genetic susceptibility and molecular characteristics can differ from aggregated global data and must be understood locally to optimize lung cancer care.

Across the study period, testing shifted decisively from single-gene assays to NGS, reaching >90% of tests by 2025. This transition aligns with international guidance favoring upfront broad profiling [11], and was facilitated by Türkiye’s universal, publicly funded system [12], where phased national reimbursement for NGS panels enabled routine use by 2022–2023, setting the stage for the inflection observed in 2024–2025. This surge likely reflects broader institutional access to on-site and reference NGS, the maturation of the national social security reimbursement framework. Center-specific policy data were not collected, however, so these inferences should be interpreted cautiously. The resulting expansion of NGS made comprehensive genotyping and PD-L1 assessment available to most patients with mNSCLC, providing the necessary foundation for rational selection of targeted therapies and immunotherapies. Future work should evaluate whether widespread testing translates into better outcomes and equitable delivery of targeted therapies.

Overall, our driver profile broadly mirrors global experience with local nuances. As expected, *EGFR* mutations (16%) clustered among never-smokers, women, and non-squamous histology; the overall prevalence, consistent with prior Turkish reports [13,14], is lower than in East Asian populations yet comparable to Western cohorts [15]. Notably, *EGFR* was detected in 6% of tumors diagnosed as SCC, above typical Western estimates (1.9–5%) [16,17] but remaining below East Asian estimates (14–18%) [18,19,20], suggesting an intermediate prevalence typical Western and East Asian series. Because many diagnoses relied on small biopsies without central review, underrecognition of adenosquamous components and classification challenges are credible contributors; indeed, the 2021 WHO guidance cautions against labeling NSCLC as “squamous” on small samples without adequate morphologic and immunohistochemical support [21]. Accordingly, these observations are hypothesis-generating and warrant confirmation in prospective, centrally reviewed cohorts. Importantly, they also carry a practical message: molecular testing should not be omitted in SCC. At minimum, reflex testing (or NGS when feasible) is warranted in SCCs with clinical or pathologic “red flags” (e.g., never- or light-smoking history, female sex, younger age, ambiguous morphology/limited tissue) to avoid missing actionable alterations and to ensure access to targeted therapies [4]. Other drivers in our cohort, *ALK* rearrangements (5.0%), *KRAS* G12C (2.6%), and *BRAF* V600E (3.2%), were observed at expected frequencies [22,23] and *RET* rearrangements were not detected, potentially reflecting true rarity and also limits of testing coverage.

Our co-mutation findings carry practical implications. *TP53* was the most frequent concomitant alteration across drivers, present in 40.5% of *EGFR*-mutant tumors. Although we lacked treatment-level detail, accumulating evidence suggests that *TP53* co-mutation in *EGFR*-mutant NSCLC portends inferior outcomes on *EGFR* TKIs [24,25,26]. In *KRAS*-mutant disease, co-alterations in *STK11/LKB1* and/or *KEAP1* demarcate subsets with distinct biology and attenuated benefit from PD-1/PD-L1 blockade [27,28]. Although our sample size precluded survival analysis, the ability to detect these immunotherapy-resistant profiles allows clinicians to better contextualize heterogeneous outcomes and consider prioritizing combination strategies or clinical trial referral over single-agent immunotherapy for these high-risk subgroups.

We investigated associations between PD-L1 expression and specific oncogenic drivers. Tumors with *EGFR* mutations and *ROS1* rearrangements showed numerically lower PD-L1 positivity, consistent with prior reports of modest single-agent ICI activity in these subgroups [29,30,31], However, after multivariable adjustment and Benjamini–Hochberg FDR correction, no driver alteration reached statistical significance. Given small subgroup sizes, PD-L1 missingness, and heterogeneous IHC platforms, these signals should be considered hypothesis-generating and warrant confirmation in larger, prospectively collected, methodologically standardized cohorts. Taken together, PD-L1 expression alone remains a heterogeneous and complex biomarker that should be interpreted with caution in oncogene-addicted NSCLC.

**Figure 5:**
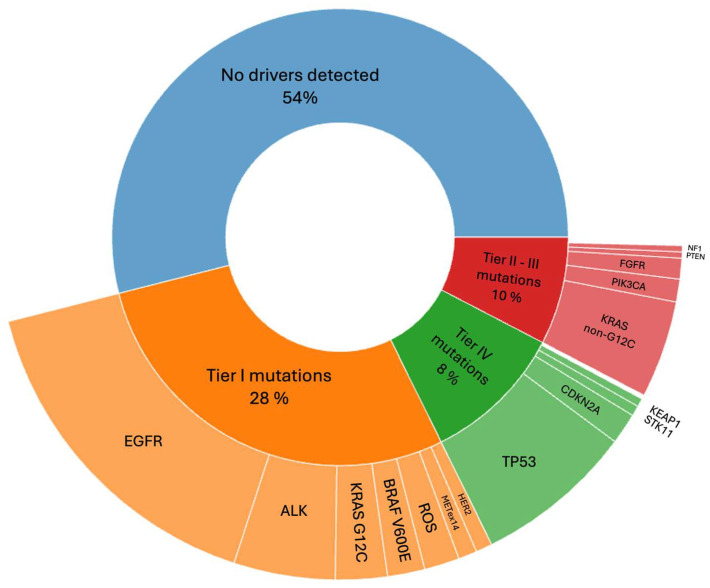
Sunburst chart illustrating the genomic actionability landscape stratified by ESMO-ESCAT evidence levels. The inner ring represents the distribution of patients according to clinical actionability tiers, while the outer ring details the specific genomic alterations within each tier. Inner Ring (Clinical Actionability): Patients are classified into Standard Actionable (Tier I), Potentially Actionable (Tier II/III), Prognostic Only, and No Driver Detected groups based on the ESMO-ESCAT framework [32] and current guidelines [11]. ALK: anaplastic lymphoma kinase, BRAF: B-Raf proto-oncogene, CDKN2A: cyclin-dependent kinase inhibitor 2A, EGFR: epidermal growth factor receptor, FGFR: fibroblast growth factor receptor, HER2: human epidermal growth factor receptor 2, KEAP1: Kelch-like ECH-associated protein 1, KRAS: Kirsten rat sarcoma viral oncogene homolog, METex14: mesenchymal–epithelial transition exon 14 skipping mutation, NF1: Neurofibromatosis type 1, PIK3CA: phosphatidylinositol-4,5-bisphosphate 3-kinase catalytic subunit alpha, PTEN: Phosphatase and TENsin homolog, ROS1: ROS proto-oncogene 1, STK11: serine/threonine kinase 11, TP53: Tumor protein p53.

While single-gene testing historically captured *EGFR* and *ALK*, the inflection toward broad-panel NGS (reaching >90% by 2025) has revealed a more complex genomic landscape. As visualized in Figure 5, Tier I Standard Actionable drivers [32] were identified in 28.3% of patients and Tier II/III alterations in a further 7.6%, meaning that approximately one-third of patients harbor variants that either already guide therapy [11] or may become targets for emerging agents and clinical trials. These frequencies, combined with the high uptake of panel-based testing, support reflex broad-panel NGS at the time of pathological diagnosis of metastatic NSCLC rather than after empiric systemic therapy, so that eligible patients are identified early enough to receive genotype-matched treatment or trial referral.

Within this landscape, a distinct subgroup of patients harbors Tier II/III (potentially actionable) alterations [32], such as *PIK3CA*, *PTEN*, and *FGFR*, alongside purely prognostic markers. Although these alterations are not currently part of the standard treatment algorithm for NSCLC [11], their identification carries significant real-world utility. Consistent with reports on the prevalence and clinical implications of *PIK3CA* aberrations across cancer types [33] and the emerging role of *PTEN* alterations in NSCLC biology [34], these variants help refine prognostic expectations and may signal mechanisms of resistance to other therapies. In practice, detecting *PIK3CA* or *PTEN* alterations may not trigger a specific approved therapy today, but it can flag patients for closer follow-up, inform discussions about likely disease course, and support consideration of clinical trial enrollment.

Ultimately, the value of molecular profiling lies in its ability to enable genotype-matched therapies. Broad overviews of cancer treatment and future directions emphasize that newer generations of targeted agents will increasingly require robust upfront genomic characterization [35]. As NGS becomes embedded in routine care in Türkiye, aligning clinical trial design and future reimbursement decisions with these real-world prevalence data may help ensure that diagnostic advances can translate into therapeutic benefit.

One area that merits deeper exploration is the influence of tobacco exposure. Türkiye has among the highest rates of tobacco consumption globally, particularly among men, with current smoking prevalence exceeding 40% and substantial rates of secondhand smoke exposure [8]. Our cohort mirrored this trend, with over 80% of patients having a smoking history. Interestingly, smoking status was strongly associated with the mutational profile—*EGFR*, *ALK*, and *ROS1* alterations were more frequent in never-smokers, while *KRAS* G12C was more common in smokers. These findings reinforce the need to pair comprehensive molecular testing with routine, structured smoking-cessation support as a standard component of lung cancer care.

The retrospective nature of the study introduces potential biases, including missing data, and variable sample quality. Testing platforms varied across centers (panel size, nominal depth/LOD, bioinformatics pipelines) and PD-L1 assays (clone/platform), which we harmonized at the alteration-present/absent level; nevertheless, assay variability may contribute to between-group differences. Additionally, treatment response and resistance mechanisms were not captured, which limits causal inference for survival differences and the prediction of immunotherapy benefit by genotype. PD-L1 testing was not available in all cases, and co-mutation analysis was restricted to the subset of patients who underwent NGS. Finally, the lack of detailed socioeconomic, occupational, and environmental exposure data may obscure important modifiers of molecular patterns as well as potential histologic misclassification. Nevertheless, key strengths include a large sample spanning seven regions, contemporaneous practice patterns that capture the real-world rollout of NGS, and integrated reporting of drivers, PD-L1, and co-mutations. Importantly, in the absence of a Surveillance, Epidemiology, and End Results Program (SEER)-like nationwide cancer genomics registry in Türkiye, this multicenter study provides rare, system-level visibility into molecular testing patterns and genomic profiles across diverse institutions and regions, thereby helping to fill a critical evidence gap.

## 5. Conclusions

This study enhances our understanding of the genomic landscape of mNSCLC in a middle-income country with a high lung cancer burden. It underscores the feasibility of implementing advanced diagnostics in a publicly funded healthcare setting and highlights the heterogeneity of actionable mutations across demographic and histologic subgroups. Future prospective studies integrating treatment outcomes, resistance mechanisms, and real-world effectiveness of targeted therapies are warranted to further optimize lung cancer care in Türkiye and similar settings.

## Figures and Tables

**Table 1:** Comparison of clinical and pathological characteristics according to driver mutation status in mNSCLC.

		*EGFR* Mutation			*ALK* Fusion			*ROS1* Fusion			*BRAF* V600E mutation			*KRAS* G12C mutation		
Variable	All Patients (n = 1023)	Mutant (n = 164)	Wild (n = 856)	*p*	Fusion Positive (n = 51)	Wild (n = 943)	*p*	Fusion Positive (n = 18)	Wild (n = 909)	*p*	Mutant (n = 22)	Wild (n = 671)	*p*-Value	Mutant (n = 26)	Wild (n = 964)	*p*
Mean age, years (SD)	64.06 ± 9.79	63.31 ± 11.46	64.21 ± 9.45	0.282	59.69 ± 11.66	64.22 ± 9.57	0.001	63.97 ± 9.62	58.83 ± 10.10	0.025 *	66.91 ± 10.69	63.90 ± 9.44	0.143 *	63.93 ± 9.76	64.62 ± 8.30	0.724 *
Age ≥ 65, n (%)	505 (49.4%)	70 (42.7%)	433 (50.6%)	0.064	17 (33.3%)	470 (49.8%)	0.022	5 (27.8%)	446 (49.1%)	0.074 *	11 (50.0%)	327 (48.7%)		15 (57.7%)	15 (57.7%)	0.363 *
Sex, n (%)				<0.001			<0.001			0.982 *			0.700 *			0.133 *
Female	241 (23.6%)	90 (37.7%)	149 (17.4%)		27 (52.9%)	205 (21.4%)		4 (22.2%)	204 (22.4%)		4 (18.2%)	145 (21.6%)		3 (11.5%)	234 (24.3%)	
Male	782 (76.4%)	74 (62.3%)	707 (82.6%)		24 (47.1%)	738 (78.3%)		14 (77.8%)	705 (77.6%)		18 (81.8%)	526 (78.4%)		23 (88.5%)	730 (75.7%)	
Smoking status, n(%) (n = 958)				<0.001			<0.001			0.462 *			0.077 *			0.635 *
Never-smoker	173 (18.1%)	78 (51.0%)	94 (11.7%)		24 (54.5%)	147 (16.6%)		2 (11.8%)	158 (18.5%)		3 (15.0%)	113 (17.9%)		3 (11.5%)	168 (18.5%)	
Active smoker	464 (48.4%)	37 (24.2%)	425 (53.0%)		11 (25.0%)	442 (49.8%)		11 (64.7%)	423 (49.6%)		7 (35.0%)	348 (55.0%)		13 (50.0%)	438 (48.3%)	
Ex-smoker	321 (33.5%)	38 (24.8%)	283 (35.3%)		9 (20.5%)	298 (33.6%)		4 (23.5%)	272 (31.9%)		10 (50.0%)	172 (27.2%)		10 (38.5%)	300 (33.1%)	
Histology, n (%)				<0.001			<0.001			0.012 *			0.214 *			0.082 *
Non-squamous carcinoma	777 (76.0%)	148 (90.2%)	626 (73.1%)		49 (96.1%)	705 (74.8%)		671 (73.8%)	18 (100.0%)		18 (81.8%)	466 (69.4%)		24 (92.3%)	753 (78.1%)	
Squamous cell carcinoma	246 (24.0%)	16 (9.8%)	230 (26.9%)		2 (3.9%)	238 (25.2%)		238 (26.2%)	0 (0.0%)		4 (18.2%)	205 (30.6%)		2 (7.7%)	211 (21.9%)	
PD-L1 TPS ≥ 1% (n = 601), n (%)	314 (52.2%)	38 (42.7%)	276 (54.2%)	0.045	13 (52.0%)	298 (52.6%)	0.956	2 (18.2%)	304 (53.8%)	0.019 *	6 (46.2%)	245 (52.7%)	0.642 *	8 (40.0%)	293 (52.5%)	0.271 *
PD-L1 TPS ≥ 50% (n = 601), n (%)	107 (17.8%)	9 (8.4%)	98 (19.3%)	0.056	7 (28.0%)	99 (17.5%)	0.311	104 (18.4%)	1 (9.1%)	0.062 *	2 (15.4%)	77 (16.6%)	0.893 *	2 (10.0%)	98 (17.6%)	0.495 *

*ALK*: anaplastic lymphoma kinase, *BRAF*: B-Raf proto-oncogene, *EGFR*: epidermal growth factor receptor; *KRAS:* Kirsten rat sarcoma viral oncogene homolog; PD-L1: programmed death-ligand 1; *ROS1*: ROS proto-oncogene 1. * *p*-values for *ROS1* fusion, *KRAS*, and *BRAF* mutations should be interpreted with caution due to small subgroup sizes.

## Data Availability

Data is not publicly available due to confidentiality and institutional privacy regulations. De-identified data underlying the findings are available to the corresponding author on reasonable request.

## Supplementary Materials

The following supporting information can be downloaded at: https://www.mdpi.com/article/10.3390/genes16121446/s1, Table S1. Center-level NGS panel specifications and PD-L1 clones with adoption timelines. Table S2. Center-level year-by-year distribution of testing method (Single-gene vs NGS). Table S3: Association of oncogenic drivers with PD-L1 positivity (TPS ≥ 1%): multivariable logistic regression with FDR correction adjusted for age, sex, smoking status, histologic subtype, and PD-L1 assay.
