# Supplementary material for: Bridging East and West: Real-World Clinicogenomic Landscape of Metastatic NSCLC in Türkiye
Source: Genes (Basel). 2025 Dec 3;16(12):1446. doi: 10.3390/genes16121446 (PMC12733280; doi:10.3390/genes16121446)
Supplement: Supplementary file 1 [file genes-16-01446-s001.zip › genes-4002977-supplementary.pdf]

Center	NGS Panel	NGS Gene Coverage	NGS Assay Vendor	PD-L1 Clone
1	Qiaseq Targeted DNA Custom Panel	AKT1, ALK, APC, ARID1A, ATM, ATRX, BRAF, BRCA1, BRCA2, BRIP1, CDH1, CDKN2A, CHEK2, CTNNB1, EGFR, ERBB2, ERBB4, ESR1, EZH2, FBXW7, FGFR1, FGFR2, FGFR3, FLT3, HNF1A, HRAS, IDH1, IDH2, JAK2, KEAP1, KIT, KRAS, LMNA, MAP2K2, MET, MLH1, MPL, MSH6, MYCN, NCOA4, NF1, NOTCH1, NPM1, NRAS, NTRK1, NTRK3, PAX8, PDGFRA, PIK3CA, POLE, PPARG, PTEN, RAB7A, RAD51C, RAD51D, RB1, RET, ROS1, SEPT14, SLC34A2, SLC45A3, SMARCB1, STK11, TACC3, TMPRSS2, TP53, TPM3<br>The reading depth is 1000X.	QIAGEN, Illumina, Thermo Fisher Qubit	Ventana SP263
2	Somatic Lung Cancer Panel	AKT1, ALK, BRAF, CDK4, CDK6, DDR2, EGFR, ERBB2, ERBB4, ESR1, FGFR1, GAPDH, IDH1, IDH2, KIT, KRAS, MAP2K1, MET, MYD88, NRAS, NTRK1, PDGFRA, PIK3CA, PTEN, RICTOR, ROS1, SOD2, STK11, TERT, TP53<br>The reading depth is ≥250x.	Element Biosciences – AVITI	Dako 22C3
3	Qiaseq Targeted DNA Custom Panel	BRAF, EGFR, KRAS, NRAS, AKT1, ALK, DDR2, ERBB2/HER2, ESR1, FGFR1, KIT, MAP2K1, MET, NTRK1, PDGFRA, PIK3CA, PTEN, RICTOR, ROS1<br>The reading depth is >100x.	QIAGEN, Illumina	Dako 22C3
4	Qiaseq Targeted DNA Custom Panel	ABL1, AKT1, AKT2, AKT3, ALK, APC, ARID1A, ATM, ATRX, BCOR, BRAF, BRCA1, BRCA2, BRIP1, CDH1, CDK4, CDKN2A, CSF1R, CTNNB1, DDR2, DICER1, DNMT3A, EGFR, ERBB2, ERBB3, ERCC2, ERCC3, ERCC4, ESR1, EZH2, FANCA, FAT1, FBXW7, FGFR1, FGFR2, FGFR3, FLT3, FOXL2, GNA11, GNAQ, GNAS, HNF1A, HRAS, IDH1, IDH2, JAK1, JAK2, JAK3, KEAP1, KIT, KRAS, MAP2K1, MAP2K2, MET, MLH1, MPL, MSH2, MSH6, MTOR, NF1, NF2, NFE2L2, NOTCH1, NPM1, NRAS, NTRK2, NTRK3, PDGFRA, PIK3CA, PLAG1, PMS2, POLE, PTCH1, PTEN, PTPN11, PTPRD, RAD50, RAD51C, RAD51D, RAF1, RB1, RET, ELOC, SETD2, SMAD4, SMARCB1, SMO, SRC, STK11, TERT, TP53, TSC1, TSC2, VHL, MSI<br>The reading depth is >100X.	QIAGEN, Element Biosciences	Dako 22C3
5	Qiaseq Targeted DNA Custom Panel	ABL1, ALK, AKT1, AKT2, AKT3, ATM, APC, ARID1A, ATRX, BARD1, BRAF, BRIP1, CDK4, CDK12, CDKN2A, CDKN2B, CHEK1, CHEK2, CTNNB1. DOR2, EGFR. ERBB2, ERBB4, FGFR1, FGFR2, FGFR3, HRAS, KEAP1, KIT, KRAS, MAP2K1, MET, MLH1, MLH3, MSH2, MSH3, MSH6, NRAS, NKX2-1, PDGFRA, PIK3CA. PMS2, PTEN, RB1, RET, ROS1, RICTOR, SMARCA2, SMARCA4, SMARCB1, STK11, TERT, TP53, TSC2<br>The reading depth is ≥250X.	Element Biosciences AVITI	SP263
6	QIAseq New Solid Custom Panel	AKT1, AKT2, AKT3, ALK, APC, ARID1A, ATM, ATRX, BAP1, BRAF, BRCA1, BRCA2, CDKN2A, CDKN2B, CHEK1, CHEK2, CTNNB1, DDR2, DICER1, EGFR, ERBB2, ERBB3, ERBB4, ESR1, FBX011 (MSH6), FGFR1, FGFR2, FGFR3, FGFR4, FLT3, GNAS, HRAS, HSPH1, IDH1, KEAP1, KIT, KRAS, MAP2K1, MAP2K2, MET, MSH2, MSH6, MTOR, NF1, NOTCH1, NOTCH2, NOTCH3, NOTCH4, NRAS, NTRK1, NTRK2, NTRK3, PDGFRA, PIK3CA, POLE, PTEN, RB1, REEP5, RET, RICTOR, ROS1, SETD2, SLC7A8, SMARCA4, SMARCB1, STK11, STT3A, TERT, TP53, ZNF2 The reading depth is >100X.	QIAGEN	Before 2023 SP263<br>Since 2023 Dako 22C3
7	QIAseq New Solid Custom Panel	ABL1, AKT1, AKT2, AKT3, ALK, APC, ARID1A, ATM, ATRX, BCOR, BRAF, BRCA1, BRCA2, BRIP1, CDH1, CDK4, CDKN2A, CSF1R, CTNNB1, DDR2, DICER1, DNMT3A, EGFR, ERBB2, ERBB3, ERCC2, ERCC3, ERCC4, ESR1, EZH2, FANCA, FAT1, FBXW7, FGFR1, FGFR2, FGFR3, FLT3, FOXL2, GNA11, GNAQ, GNAS, HNF1A, HRAS, IDH1, IDH2, JAK1, JAK2, JAK3, KEAP1, KIT, KRAS, MAP2K1, MAP2K2, MET, MLH1, MPL, MSH2, MSH6, MTOR, NF1, NF2, NFE2L2, NOTCH1, NPM1, NRAS, NTRK2, NTRK3, PDGFRA, PIK3CA, PLAG1, PMS2, POLE, PTCH1, PTEN, PTPN11, PTPRD, RAD50, RAD51C, RAD51D, RAF1, RB1, RET, ELOC, SETD2, SMAD4, SMARCB1, SMO, SRC, STK11, TERT, TP53, TSC1, TSC2, VHL<br>The reading depth is >500X.	QIAGEN	Ventana SP263

Supplementary Table S1. Center-level NGS panel specifications and PD-L1 clones with adoption timelines

Center	Method	2025	2024	2023	2022	2021	2020	≤2020
**1**	**Single-gene**	0.0%	23.5%	94.4%	87.5%	95.6%	97.6%	98.9%
	**NGS**	100.0%	76.5%	5.6%	12.5%	4.4%	2.4%	1.1%
**2**	**Single-gene**	—	—	—	—	—	—	—
	**NGS**	100.0%	100.0%	100.0%	100.0%	100.0%	100.0%	100.0%
**3**	**Single-gene**	50.0%	62.5%	92.1%	94.7%	—	—	—
	**NGS**	50.0%	37.5%	7.9%	5.3%	—	—	—
**4**	**Single-gene**	0.0%	0.0%	0.0%	25.0%	—	—	100.0%
	**NGS**	100.0%	100.0%	100.0%	75.0%	—	—	0.0%
**5**	**Single-gene**	0.0%	5.3%	4.3%	4.2%	0.0%	33.3%	66.7%
	**NGS**	100.0%	94.7%	95.7%	95.8%	100.0%	66.7%	33.3%
**6**	**Single-gene**	100.0%	81.8%	72.7%	50.0%	—	—	100.0%
	**NGS**	0.0%	18.2%	27.3%	50.0%	—	—	0.0%
**7**	**Single-gene**	—	—	—	—	—	—	—
	**NGS**	—	100.0%	100.0%	100.0%	100.0%	100.0%	100.0%
**Total**	**Single-gene**	8.3%	18.3%	45.2%	44.9%	44.8%	80.8%	94.8%
	**NGS**	91.7%	81.7%	54.8%	55.1%	55.2%	19.2%	5.2%

Supplementary Table S2. Center-level year-by-year distribution of testing method (Single-gene vs NGS)

Driver	HR	95% CI (Lower–Upper)	p	FDR q (BH)
**EGFR**	0.688	0.403 – 1.174	0.170	0.425
**ALK**	0.818	0.324 – 2.067	0.671	0.839
**ROS1**	0.222	0.047 – 1.061	0.059	0.295
**BRAF**	1.063	0.333 – 3.396	0.918	0.918
**KRAS G12C**	0.659	0.262 – 1.660	0.377	0.628

Supplementary Table S3: Association of oncogenic drivers with PD-L1 positivity (TPS ≥1%): multivariable logistic regression with FDR correction adjusted for age, sex, smoking status, histologic subtype, and PD-L1 assay
